# Three-dimensional collagen-based scaffold model to study the microenvironment and drug-resistance mechanisms of oropharyngeal squamous cell carcinomas

**DOI:** 10.20892/j.issn.2095-3941.2020.0482

**Published:** 2021-06-15

**Authors:** Giacomo Miserocchi, Claudia Cocchi, Alessandro De Vita, Chiara Liverani, Chiara Spadazzi, Sebastiano Calpona, Giandomenico Di Menna, Massimo Bassi, Giuseppe Meccariello, Giovanni De Luca, Angelo Campobassi, Maria Maddalena Tumedei, Alberto Bongiovanni, Valentina Fausti, Franco Cotelli, Toni Ibrahim, Laura Mercatali

**Affiliations:** 1 Osteoncology and Rare Tumors Center, IRCCS Istituto Romagnolo per lo Studio dei Tumori (IRST) “Dino Amadori”, Meldola 47014, Italy; 2 Maxillofacial Surgery Unit, Bufalini Hospital, Cesena 47521, Italy; 3 Department of Head-Neck Surgery, Otolaryngology, Head-Neck and Oral Surgery Unit, Morgagni Pierantoni Hospital, Forlì 47121, Italy; 4 Pathology Unit, “Bufalini” Hospital, AUSL Romagna, Cesena 47521, Italy; 5 Biosciences Laboratory, IRCCS Istituto Romagnolo per lo Studio dei Tumori (IRST) “Dino Amadori”, Meldola 47014, Italy; 6 Department of Biosciences, Università degli Studi di Milano, Milan 20133, Italy

**Keywords:** Oropharyngeal squamous cell carcinoma, collagen, biomimetic scaffold, zebrafish, drug-resistance, primary culture

## Abstract

**Objective:**

Squamous cell carcinoma (SCC) represents the most common histotype of all head and neck malignancies and includes oropharyngeal squamous cell carcinoma (OSCC), a tumor associated with different clinical outcomes and linked to human papilloma virus (HPV) status. Translational research has few available *in vitro* models with which to study the different pathophysiological behavior of OSCCs. The present study proposes a 3-dimensional (3D) biomimetic collagen-based scaffold to mimic the tumor microenvironment and the crosstalk between the extracellular matrix (ECM) and cancer cells.

**Methods:**

We compared the phenotypic and genetic features of HPV-positive and HPV-negative OSCC cell lines cultured on common monolayer supports and on scaffolds. We also explored cancer cell adaptation to the 3D microenvironment and its impact on the efficacy of drugs tested on cell lines and primary cultures.

**Results:**

HPV-positive and HPV-negative cell lines were successfully grown in the 3D model and displayed different collagen fiber organization. The 3D cultures induced an increased expression of markers related to epithelial–mesenchymal transition (EMT) and to matrix interactions and showed different migration behavior, as confirmed by zebrafish embryo xenografts. The expression of hypoxia-inducible factor 1α (1α) and glycolysis markers were indicative of the development of a hypoxic microenvironment inside the scaffold area. Furthermore, the 3D cultures activated drug-resistance signaling pathways in both cell lines and primary cultures.

**Conclusions:**

Our results suggest that collagen-based scaffolds could be a suitable model for the reproduction of the pathophysiological features of OSCCs. Moreover, 3D architecture appears capable of inducing drug-resistance processes that can be studied to better our understanding of the different clinical outcomes of HPV-positive and HPV-negative patients with OSCCs.

## Introduction

Head and neck carcinomas (HNCs) represent the sixth most common non-skin cancer worldwide^1^, with an annual incidence of about 400,000 new cases and a 50% mortality rate^2,3^. Squamous cell carcinomas (SCCs) represent approximately 90% of all HNCs^4^. Although the primary risk factors are alcohol abuse and tobacco, human papilloma virus (HPV) has now been linked to the development of some tumor types^5^. HPV-positive SCCs arise mainly in the oropharynx and have different pathological and clinical characteristics with respect to HPV-negative tumors^6–8^. Oropharyngeal squamous cell carcinomas (OSCCs) show a more favorable clinical outcome than alcohol- and tobacco-related SCCs^9^. Standard treatment options for SCCs include radiation, surgery, chemotherapy, targeted therapies, and immunotherapy. In OSCCs, chemotherapy is often used in advanced stages of the disease. 5-fluorouracil (5-FU) and platinum-based drugs [cisplatin (CIS) and carboplatin] represent the most widely used chemotherapeutic agents, whereas taxol-based treatments are preferred in the metastatic setting^10^. The only targeted therapy approved for OSCCs, regardless of HPV status, is cetuximab (CETU), an epidermal growth factor receptor (EGFR)-targeting monoclonal antibody used alone or in combination with chemotherapy^11^. Immune checkpoint inhibitors show great promise for the treatment of HNCs, and several clinical trials and preclinical studies of anti-cytotoxic T-lymphocyte–associated antigen 4, anti-programmed death 1, anti-programmed death ligand 1, and novel immunotherapeutic drugs are currently ongoing to identify new treatment options^12^.

Despite these innovative therapeutic approaches, disease relapse and drug resistance remain a challenge in OSCCs. Response to treatment is influenced by many factors, including interaction with the extracellular matrix (ECM)^13^. Like other cancers, SCCs are dynamic masses that remodel the 3-dimensional (3D) ECM structure and interact constantly with stromal components^14^. The ECM has a fiber network that provides physical support and is also involved in regulating numerous cellular processes^15^. For example, during cancer progression, the association between integrins and ECM components induces cell adhesion-mediated drug resistance^16^. Integrin signaling supports ECM biosynthesis, increases collagen fiber crosslinking, and sustains chemotaxis-driven cell invasion^17^. Given that the reproduction of these tumor microenvironment features is virtually impossible in monolayer models, 3D cultures have been developed in an attempt to mimic the architecture of the tumor niche^18–20^.

In the present study we investigated, as far as we know, for the first time, the impact of a 3D biomimetic collagen-based scaffold on the phenotypic and genotypic features of OSCC cells. We also evaluated the role of scaffold architecture in the response of cell lines and patient-derived primary cultures to standard treatments.

## Materials and methods

### Collagen-based scaffold synthesis

Collagen scaffolds were synthesized using chemicals purchased from Sigma-Aldrich (St. Louis, MO, USA). Bovine type I collagen (1 wt%) was suspended and precipitated to pH 5.5. The collagen was crosslinked with 1.4-butanediol diglycidyl ether (1 wt%) to control porosity and stabilize the collagen matrix. A freeze-drying process was used to generate the final monolithic scaffold. An established freezing and heating ramp yielded scaffolds with optimal levels of pore interconnectivity, porosity, and size (as previously reported)^21^. Obtained scaffolds were immersed in 70% ethanol for 1 h for complete sterilization and followed by 3 washes of 5′ each in sterile Dulbecco phosphate-buffered saline (Life Technologies, Carlsbad, CA, USA).

### Two-dimensional (2D) and 3D cell cultures and reagents

UPCI:SCC090 OSCC cell line was purchased from American Type Culture Collection (Rockville, MD, USA), and UM-SCC6 HPV-negative OSCC cell line was obtained from MD Millipore (Merck SpA, Darmstadt, Germany). UPCI:SCC090 cells were maintained in Eagle’s minimum essential medium (ATCC, Rockville, MD, USA) supplemented with 1% of l-glutamine (PAA, Piscataway, NJ, USA) and 10% of fetal bovine serum (FBS; Euroclone, Milan, Italy). UM-SCC6 cell line was cultured in Dulbecco’s modified Eagle’s medium (DMEM) High Medium (Euroclone) supplemented with 1% penicillin/streptomycin (PAA), 1% non-essential amino acids (Euroclone), and 10% FBS. Cells were counted and seeded in monolayer cultures or in collagen-based scaffolds^13^. The growth curves were generated on 2D and 3D cultures (concentration of 3.5 × 10^6^ cells for scaffold) through MTT tests (Sigma-Aldrich) at 24 h, 72 h, and 5 days. All cell lines were maintained in a 5% CO_2_ atmosphere at 37 °C.

### Immunohistochemical and immunofluorescence staining

For staining protocols, collagen-based scaffolds were embedded in paraffin, using cryomold (25 × 20 × 5 mm). To evaluate the culture’s morphological features, 5-µm-thick slides were used for staining. Slides were hydrated and stained with hematoxylin and eosin (H&E; Sigma-Aldrich) or with phalloidin and 4′,6-diamidino-2-phenylindole (Invitrogen, Carlsbad, CA, USA).

Hypoxia-inducible factor 1α (HIF-1α) expression was determined in 5-µm sections and on cells cytospinned onto glass slides with Ventana BenchMark ULTRA system (Ventana Medical Systems, Tucson, AZ, USA) according to the manufacturer’s instructions. The samples were stained with rabbit monoclonal anti-HIF-1α antibody (1:100, ab51608) and glucose transporter 1 (GLUT-1, 1:1,000, ab115730) (both from Abcam, Cambridge, UK). Counterstaining was performed with hematoxylin. Stained slides were analyzed using an inverted microscope (Axioskop; Carl Zeiss, Gottïngen, Germany). Images of phalloidin-stained samples images were acquired by a confocal microscope and analyzed with the NIS Elements software (both from Nikon Corporation, Tokyo, Japan).

### Gene expression analysis

mRNA extraction and quantitative polymerase chain reaction (qPCR) assay were performed as previously described^21^. 2D culture cells were collected by trypsinization and resuspended in TRIzol reagent (Invitrogen); meanwhile, 3D culture cells were processed with TRIzol directly on the scaffolds. All specimens were conserved at −80 °C. RNA (500 ng) was retro-transcribed to obtain cDNA using iScript cDNA Synthesis Kit (BioRad, Hercules, CA, USA). A 2× Taqman Universal PCR Master Mix and a 7,500 Real-Time PCR System (both from Applied Biosystems, Foster City, CA, USA) were used for the amplification of 2 µL of cDNA by real-time polymerase chain reaction assay. The following genes were analyzed: *CDH1*, vimentin (*VIM*), *SNAIL*, *MMP2*, *MMP9*, *LOX*, *GAPDH*, *NT5E*, *FOXM1*, *FLT1*, *ABCA3*, *CXCR4*, *TP53*, and *TGFβ*. *ACTB* and *HPRT* were used as housekeeping genes. The obtained data were normalized to the housekeeping genes with the delta-delta Ct (2-∆∆Ct) method.

### Zebrafish husbandry

Tg(fli1:EGFP) transgenic zebrafish strain was handled in compliance with local animal welfare regulations (authorization No. prot. 18311/2016; the authorization for zebrafish breeding in the IRST facility was released by the “Comune di Meldola”, 09/11/2016) and in conformity with the Directive 2010/63/EU. Fertilized eggs were collected by natural spawning and raised at 28 °C in embryo water with 0.1% methylene blue, according to Kimmel et al.^22^. Before manipulation, zebrafish embryos were anesthetized in 0.02% tricaine solution (Sigma-Aldrich).

### Tumor xenograft in zebrafish embryos

Tg(fli1:EGFP) transgenic zebrafish embryos were dechorionated at 48 h post-fertilization (hpf). Cells from 2D cultures were collected by trypsinization, whereas cells seeded on scaffolds were obtained after 2 mg/mL collagenase type I digestion (Millipore Corporation, Billerica, MA, USA) 1:1 in DMEM High Medium for 15 min at 37 °C under stirring conditions. Cells were labeled with a red fluorescent dye (CellTracker™ CM-DiI; Invitrogen) and resuspended in PBS at a concentration of 2.5 × 10^5^/µL. 300/500 cells were implanted in the sub-peridermal space of 48-hpf embryos after tricaine anesthetization. Embryos injected in the yolk sack or/and that show cancer cells in circulation were excluded. The 2 groups injected with 2D or 3D culture cells were incubated at 32 °C. At 24 h post-injection (hpi), the presence of circulating cells and micro-metastasis development was evaluated using a fluorescence stereomicroscope (Nikon SMZ 25 equipped with NIS Elements software).

### Drug sensitivity test

Drug sensitivity assays were performed on 2D and 3D cultures. Two days after seeding, the cell lines were treated with plasmatic peak concentrations of CIS, 5-FU, CETU, and gemcitabine (GEMCI) in accordance with the pharmacokinetic/clinical data for each drug. CIS was administered at a concentration of 4.1 µg/mL^23^, 5-FU at 55.44 µg/mL^24^, CETU at 130 µg/mL^25^, and GEMCI at 15.83 µg/mL^26^. After 72 h of treatment, surviving cell fractions were measured using the MTT test (Sigma-Aldrich) following the manufacturer’s protocol^27^.

### Establishment of primary cell cultures

The study involved 2 patients affected by SCCs. Patients underwent surgical treatment after having signed informed written consent. Patient-derived primary cultures were obtained from surgical specimens. The tissue samples were analyzed by an experienced pathologist, and a section of cancer tissue was transported under sterile conditions to the Biosciences Laboratory of our institute (IRST IRCCS) within 45 min of removal. Tumor samples were washed twice with PBS and minced with surgical scalpels into fragments of approximately 0.5–1 mm^3^ as previously reported^28^. Fragments were incubated in a PBS solution of 2 mg/mL collagenase type I (Millipore Corporation) 1:1 in DMEM High Medium for 15 min at 37 °C and then at room temperature for a further 15 min. The suspension was then filtered with a 100-µm sterile mesh filter (CellTrics; Partec, Münster, Germany). Single-cell suspensions were seeded in monolayer or 3D cultures and maintained at 37 °C in a 5% CO_2_-humidified atmosphere. All the experiments were performed within 2 weeks and analyzed by an experienced pathologist. The present study was approved by the IRST-Area Vasta Romagna Ethics Committee (Approval No. 4751/2015) and performed according to Good Clinical Practice standards and with the principles laid down in the Declaration of Helsinki (1964).

### Statistical analysis

Each experiment was repeated at least 3 times. Data are shown as mean ± standard deviation or mean ± standard error, as stated, with *n* indicating the number of replicates. Two-tailed Student’s t-test was used to define the differences between groups, and *P* values < 0.05 were considered significant.

## Results

### 3D collagen-based scaffold reproduces different tissue-like organizations and phenotypes

**Figure 1:**
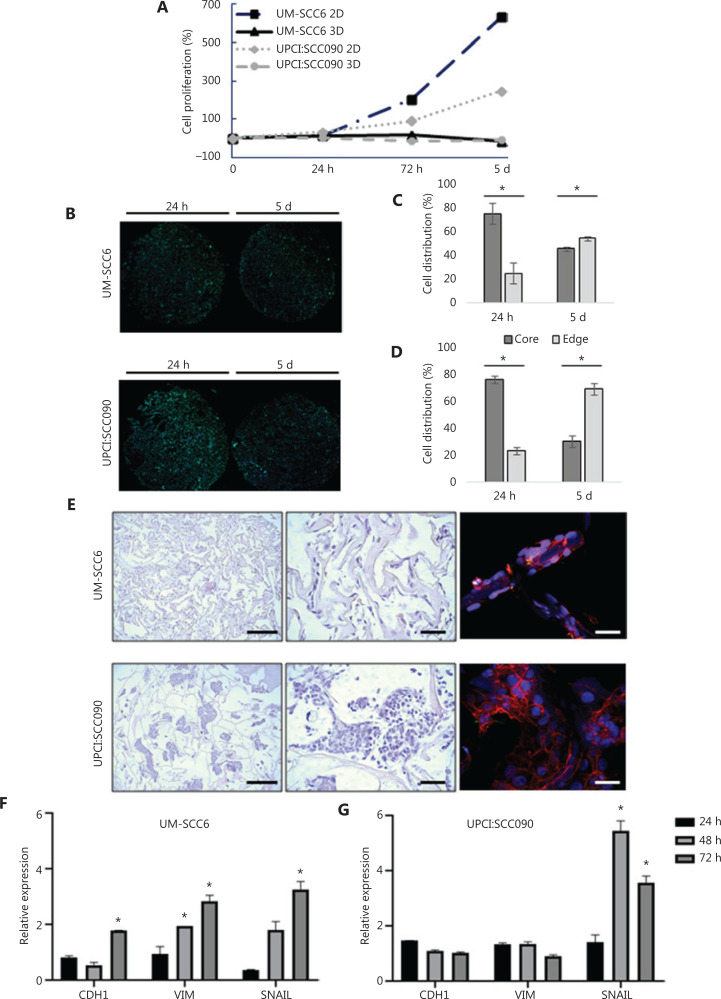
Characterization of the 3D oropharyngeal squamous cell carcinoma culture model. (A) Fold changes in percentage of cell proliferation (relative to hour 0) of UM-SCC6 and UPCI:SCC090 in monolayer (2D) and in scaffolds (3D) after 24 h, 72 h, and 5 days. (B) Whole images of histological sections of scaffold cultured with UM-SCC6 and UPCI:SCC090 on days 1 and 5. Cells are stained with DAPI (blue) and green is the collagen scaffold autofluorescence. Scale bars: 1 mm. (C, D) Cell percentage in the core and at the edge of the scaffold area. Data represent mean ± standard deviation. *P < 0.05, 2-tailed Student’s *t*-test. (E) Images of H&E-stained histological sections and confocal images of UM-SCC6 and UPCI:SCC090 scaffold cultures. From above: H&E images at 4× magnification (scale bars: 100 µm) and 20× magnification (scale bars: 50 µm); confocal images of sections stained with phalloidin in red and DAPI in blue (scale bar: 20 µm). (F, G) *CDH1*, *VIM*, and *SNAIL* relative mRNA expression in UM-SCC6 and UPCI:SCC090 within the scaffold *vs.* 2D cultures after 24, 48, and 72 h. Data represent mean ± standard deviation. *P < 0.05, 2-tailed Student’s *t*-test. 3D, 3-dimensional; 2D, 2-dimensional; H&E, hematoxylin and eosin; DAPI, 4′,6-diamidino-2-phenylindole.

The proliferation curves of UM-SCC6 HPV-negative cells and UPCI:SCC090 HPV-positive cells were analyzed by MTT assay at different time points. Monolayer cultures grew rapidly and constantly for up to 5 days post-seeding. Conversely, scaffold cultures did not show an exponential growth phase at the same time points, maintaining a constant proliferation over time in culture (**Figure 1A**). Confocal microscopy imaging was used to study the distribution and proliferation in different regions of the scaffold area (core and edge) (**Figure 1B–1D**). The cells of both lines showed a high concentration in the scaffold core 24 h post-seeding: 75% for UM-SCC6 and 76.5% for UPCI:SCC090. However, cell distribution changed after 5 days’ culture, with images showing higher cell concentrations at the edge of the scaffold area in both cell lines (54.5% for UM-SCC6 and 69.55% for UPCI:SCC090).

The cell organization in the 3D scaffold matrix of the 2 cell lines was analyzed by H&E staining and confocal imaging (**Figure 1E**). UM-SCC6 displayed a mesenchymal-like phenotype with homogeneous distribution along the collagen fibers, with low cell-to-cell contact. Conversely, UPCI:SCC090 showed a dense clustered organization with a higher mean cellularity. Confocal images reflected the migration tendency of UM-SCC6 and showed Indian filing, an aligned distribution over the collagen fibers that reflects an aggressiveness phenotype^29^. Conversely, UPCI:SCC090 images confirmed the tendency to grow in aggregates within the porous area and to display an epithelial-like morphology.

Gene expression analysis on 2D and 3D cultures revealed different gene expression modulations in the 2 cell lines (**Figure 1F and 1G**). UM-SCC6 showed a higher *CDH1* (E-cadherin) expression after 72 h culture in the 3D model (*P* = 0.01). No change was detected between the different UPCI:SCC090 cultures. The expression of 2 epithelial–mesenchymal transition (EMT)-related markers, *VIM* and *SNAIL*, was also analyzed. In UM-SCC6 cells, *VIM* and *SNAIL* mRNA levels increased constantly since 72 h in 3D culture. UPCI:SCC090 expressed similar *VIM* levels in both culture systems, but *SNAIL* expression significantly increased in the scaffold microenvironment.

### The interaction between cancer cells and collagen matrix induces an aggressive phenotype

**Figure 2:**
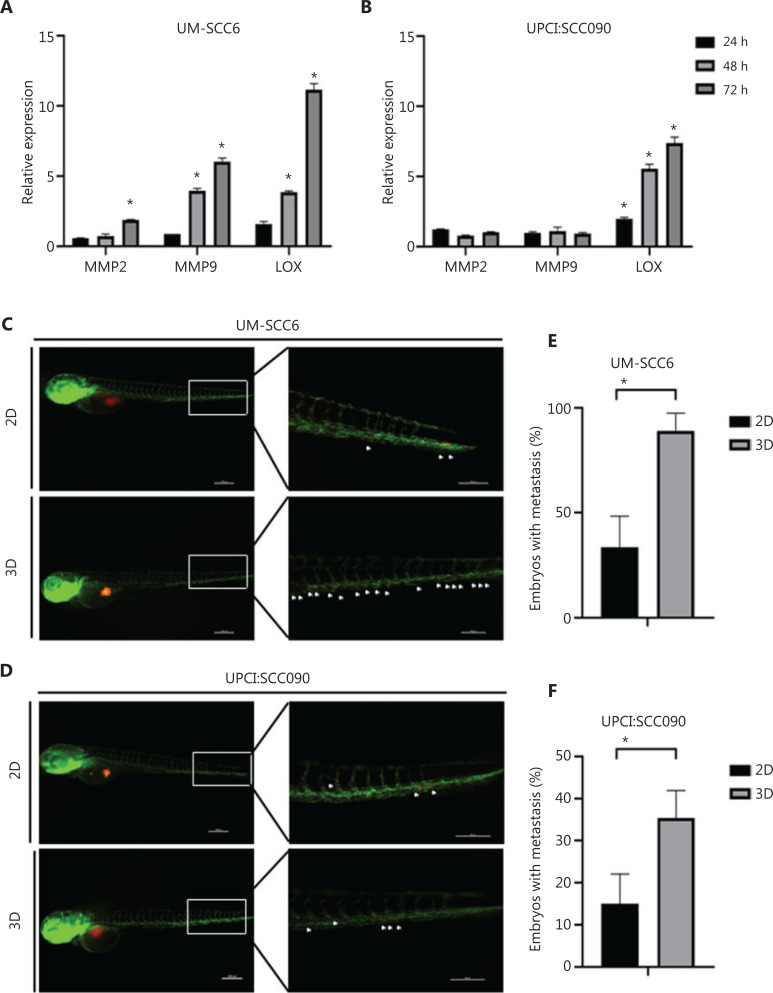
Cells cultured in the scaffold interact with collagen fiber network and display higher cell migration inside the vessels of zebrafish embryos. (A, B) UM-SCC6 and UPCI:SCC090 relative expression levels of *MMP2*, *MMP9*, and *LOX* in 3D *vs.* 2D cultures after 24 h, 48 h, and 72 h. Data represent mean ± standard deviation. *P < 0.05, 2-tailed Student’s *t*-test. (C, D) Representative pictures of Tg(fli1:EGFP) embryos injected with UM-SCC6 and UPCI:SCC090 recovered from 2D and 3D cultures and labeled with a red fluorescent dye (CM-Dil). The images show the whole embryo body and tail region details at 24 hpi. The white asterisks show single or small cell clusters. (E, F) Percentage of embryos showing distant micrometastases. Data represent mean ± standard error. *P < 0.05, 2-tailed Student’s t-test. 3D, 3-dimensional; 2D, 2-dimensional.

We analyzed 2 ECM-degrading metalloenzymes, metalloproteinase 2 and 9 (*MMP2* and *MMP9*), and lysyl oxidase (*LOX*) expression to understand how OSCC cells interact with collagen fibers^29–32^. Over time in culture, the 3D microenvironment induced the overexpression of both MMPs in UM-SCC6 (after 72 h for *MMP2*, *P* = 0.002; after 48 h and 72 h for MMP 9, *P* = 0.004 and *P* = 0.01, respectively) (**Figure 2A**). No modulation in MMP expression was observed in UPCI:SCC090 (**Figure 2B**). Conversely, *LOX* was the most upregulated marker in both cell lines cultured in 3D, with expression levels 11.15- and 7.38-fold higher than those of monolayer cultures of UM-SCC6 and UPCI:SCC090, respectively (after 48 and 72 h for UM-SCC6, *P* = 0.003 and *P* = 0.009, respectively; after 24, 48, and 72 h for UPCI:SCC090, *P* = 0.02, *P* = 0.02, and *P* = 0.01, respectively).

We used the zebrafish *in vivo* model to evaluate the phenotype of cells grown on collagen-based scaffolds and on monolayers (**Figure 2C and 2D**), confirming, as expected, the more aggressive phenotype of the former. Cells injected in the perivitelline space migrated inside the vessels and formed metastases. As previously stated, HPV-negative cells showed higher *in vitro* aggressiveness than the HPV-positive line. This result was further confirmed by the higher number of metastatic embryos at 24 hpi. Both 3D cultures showed a higher number of migrated cells inside the circulation than their monolayer culture counterparts (*P* = 0.03 for UM-SCC6 and *P* = 0.05 for UPCI:SCC090) (**Figure 2E and 2F**, respectively).

### The scaffold structure induces a hypoxic microenvironment

**Figure 3:**
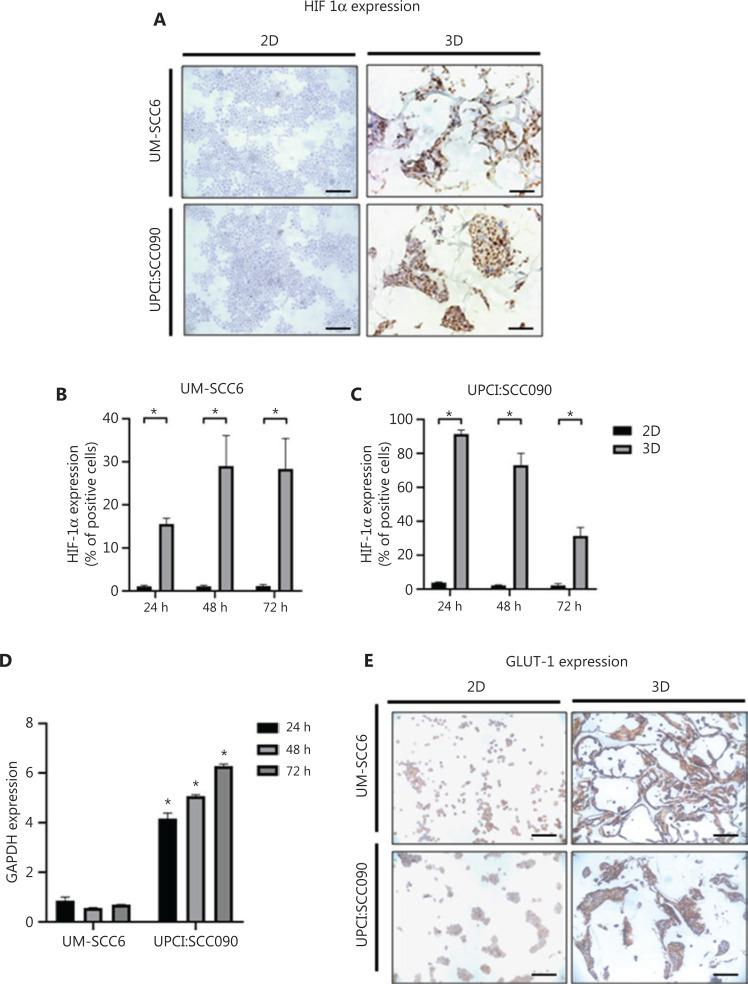
3D microenvironment induces hypoxic and glycolytic adaptation by oropharyngeal squamous cell carcinoma cells. (A) Representative images of HIF-1α-stained cytospinned cells and histological sections of 2D and 3D cultures (scale bar: 50 µm). (B, C) Percentage of HIF-1α-positive cells in 2D and 3D cultures of UM-SCC6 and UPCI:SCC090 after 24, 48, and 72 h. Data represent mean ± standard deviation. *P < 0.05, 2-tailed Student’s *t*-test. (D) GAPDH mRNA expression levels in 3D *vs.* 2D cultures after 24, 48, and 72 h. Data represent mean ± standard deviation. *P < 0.05, 2-tailed Student’s *t*-test. (E) Representative images of GLUT-1-stained cytospinned cells and histological sections of 2D and 3D cultures (scale bar: 50 µm). 3D, 3-dimensional; HIF-1α, hypoxia-inducible factor 1α; 2D, 2-dimensional; GAPDH, glyceraldehyde 3-phosphate dehydrogenase; GLUT-1, glucose transporter.

Hypoxia is one of the consequences of the development of tumors, including OSCCs^19^. 3D culture systems have proven capable of reproducing the low oxygenation of the tumor environment, closely mimicking clinical practice/a real-world setting^33,34^. We evaluated whether the oxygenation pattern changed inside the scaffold structure by analyzing the expression of the hypoxic marker HIF-1α (**Figure 3A**). Over time, the cell lines cultured in monolayers showed little or no HIF-1α positivity. Conversely, 3D cultures showed high immunohistochemical expression of HIF-1α. In particular, HPV-negative cells exhibited increased HIF-1α positivity (15.52%, 29.03%, and 28.33% of positive cells at 24, 48, and 72 h, respectively), whereas HPV-positive 3D cultures showed decreased expression of the marker during the time in culture (91.42%, 73.12%, and 31.39% of positive cells at 24, 48, and 72 h, respectively) (**Figure 3B and 3C**).

In the event of oxygen deficiency, it is known that cancer cells take advantage of glycolysis as a source of energy^35^. We determined the expression of glyceraldehyde 3-phosphate dehydrogenase (GAPDH), an enzyme involved in glycolysis processes to evaluate glycolysis stimulation in the hypoxic core. Results revealed an increment in GAPDH mRNA in UPCI:SCC090 grown on scaffolds (*P* = 0.02, *P* = 0.0002, and *P* = 0.004 at 24, 48, and 72 h, respectively), whereas UM-SCC6 maintained the same expression levels in 2D and 3D cultures (**Figure 3D**). We also measured the immunohistochemical expression of the glucose transporter (GLUT-1) to confirm glycolysis stimulation. Images displayed a higher membrane expression of GLUT-1 in both cell lines seeded in the 3D scaffold than in 2D monolayer cultures (**Figure 3E**).

### 3D scaffold induces mechanisms of drug-resistance

**Figure 4:**
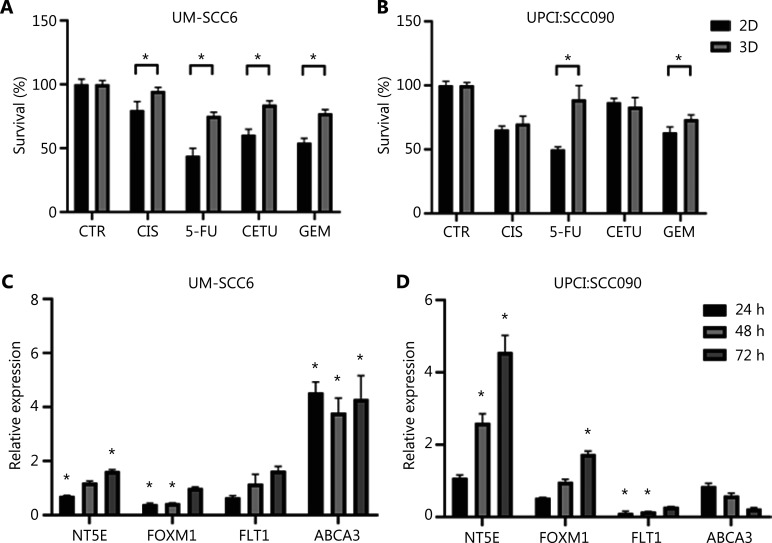
3D cultured cells acquire drug-resistance mechanisms. (A, B) Cytotoxicity analysis of UM-SCC6 and UPCI:SCC090 cell lines treated with cisplatin, 5-fluorouracil, cetuximab, and gemcitabine. Differences between 2D and 3D cultures were assessed by a 2-tailed Student’s *t*-test and accepted as significant (*) at *P* < 0.05. (C, D) *NT5E*, *FOXM1*, *FLT1*, and *ABCA3* gene expression analysis in 3D *vs.* 2D cultures after 24, 48, and 72 h. Data represent mean ± standard deviation. *P < 0.05, 2-tailed Student’s t-test. 3D, 3-dimensional; CIS, cisplatin; 5-FU, 5-fluorouracil; CETU, cetuximab; GEM, gemcitabine; 2D, 2-dimensional.

Hypothesizing that 3D collagen-based scaffolds can successfully predict drug-induced cytotoxic effects, we treated 2D and 3D cultures with drugs used in clinical practice for OSCCs CIS, 5-FU, CETU, and GEM. The plasma peak concentration was used for all drugs. UM-SCC6 displayed a low response to each treatment when seeded on scaffolds (CIS, *P* = 0.03; 5-FU, *P* = 0.0002; CETU, *P* = 0.0002; GEM, *P* = 0.0001) (**Figure 4A**). A similar behavior was observed for UPCI:SCC090 (**Figure 4B**), with the exception of a higher response to CETU in 2D cultures than in 3D models (5-FU, *P* = 0.003; GEM, *P* = 0.04).

We analyzed the expression of several genes involved in drug-resistance processes, including *NT5E*, *FOXM1*, *FLT1*, and *ABCA3* (**Figure 4C and 4D**). Findings showed higher expression levels of *NT5E* and *FOXM1* in 3D HPV-positive cells (4.57- and 1.73-fold higher expression at 72 h, respectively) and lower levels in the HPV-negative cell line. The expression levels of *FLT1* in UM-SCC6 3D cells were 1.63-fold higher than that in 2D cultures. Conversely, there was no overexpression of *FLT1* in UPCI:SCC090 in scaffold cultures. qPCR analysis revealed increased *ABCA3* expression in HPV-negative 3D cultures (4.3-fold higher than in monolayer cultures at 72 h), not observed in HPV-positive cells.

### 3D patient-derived primary cultures confirm the induction of drug resistance

**Figure 5:**
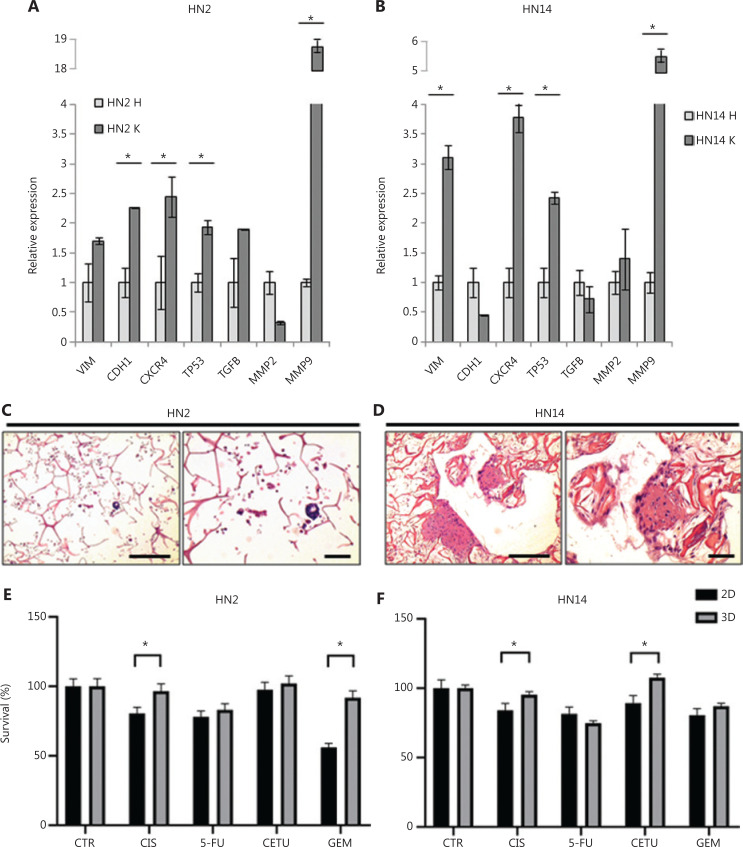
Drug-resistance mechanisms are also conserved in HNC patient-derived primary cultures. (A, B) *VIM*, *CDH1*, *CXCR4*, *TP53, TGFβ*, *MMP2*, and *MMP9* gene expression analysis in patient tumor tissue (HN2 K and HN14 K) *vs.* healthy tissue (HN2 H and HN14 H). Data represent mean ± standard deviation. *P < 0.05, 2-tailed Student’s t-test. (C, D) Images of H&E-stained histological sections of HN2 and HN14 3D primary cultures. From left: H&E images at 10× magnification (scale bars: 100 µm) and 20× magnification (scale bars: 50 µm). (E, F) Cytotoxic analysis of HN2 and HN14 primary cultures. 2D and 3D cultures were treated with CIS, 5-FU, CETU, and GEM. Differences between 2D and 3D cultures were assessed by a 2-tailed Student’s t-test and accepted as significant (*) at *P* < 0.05. HNC, head and neck carcinoma; H&E, hematoxylin and eosin; 3D, 3-dimensional; 2D, 2-dimensional; CIS, cisplatin; 5-FU, 5-fluorouracil; CETU, cetuximab; GEM, gemcitabine.

Patient-derived primary cultures were established from fresh surgery specimens obtained from 2 patients with SCCs. Patient characteristics are summarized in **Table 1**. mRNA extracted from healthy tissue (HN2 H and HN14 H) and cancer tissue (HN2 K and HN14 K) was used to detect genes involved in tumor pathological processes (**Figure 5A and 5B**). *VIM*, *CDH1*, *CXCR4*, *TP53*, *TGFβ*, *MMP2*, and *MMP9* were valuated. HN2 and HN14 tumor samples expressed higher levels of *VIM* (0.7 and 2.1, respectively), CXCR4 (1.4 and 2.8, respectively), TP53 (0.9 and 1.4, respectively), and MMP9 (17.9 and 4.7, respectively) than healthy tissue. *CDH1* and *TGFβ* were downregulated in HN2 K and upregulated in HN14 K, whereas MMP2 displayed an inverse behavior.

**Table 1:** Patient characteristics

Patient	Gender	Age (years) at surgery	Site	Size (cm)	Histological subtype	TNM staging
HN2	M	78	Oral cavity (gingiva)	8 × 4.5 × 1.1	SCC	T4aN2bM0
HN14	F	61	Oral cavity (gingiva and mandible)	8 × 7 × 5	SCC	T4aN0M0

M, male; F, female; SCC, squamous cell carcinoma.

Primary cultures preserved tumor stroma components, a key factor in maintaining the tumor microenvironment and the crosstalk between healthy and malignant cell populations^36^. H&E staining highlighted different morphology features in HN2 and HN14 primary cultures (**Figure 5C and 5D**). HN2 showed approximately 20% of cancer cells isolated or in small clusters characterized by prominent nuclei, clear central nucleolus and large eosinophilic cytoplasm. Conversely, HN14 cultures colonized the scaffold area with approximately 30% of malignant cells, isolated or aggregated in small clusters. The cells had a spindle morphology and eosinophilic cytoplasm, whereas the nuclei showed dispersed chromatin and a single, central, and clear nucleolus.

Cells seeded on scaffolds and on monolayer supports were treated with the same drugs used for cell lines (**Figure 5E and 5F**). Drug resistance was induced in 3D primary cultures respect 2D counterparts (HN2: CIS, *P* = 0.035; GEM, *P* = 0.008; HN14: CIS, *P* = 0.05; CETU, *P* = 0.02) but was less pronounced than in UM-SCC6 and UPCI:SCC090 cell lines. 5-FU was the only treatment more effective in cells in 3D HN14 cultures than in 2D HN14 cultures.

## Discussion

In recent years, the incidence of OSCCs has been increasing, especially in younger patients and in western countries^37,38^. The HPV status for these tumor types has a profound impact on patient outcome, i.e., HPV-positive OSCCs are associated with better prognosis and have different biological features with respect to HPV-negative disease^8,37,38^. A better understanding of the role played by the tumor microenvironment and of its involvement in disease pathogenesis and drug-resistance mechanisms is needed.

In the last few decades, new *in vitro* models have been developed to better mimic the structure of the tumor niche^18^. One such approach is the 3D model. An ideal cancer 3D culture should resemble the tissue architecture and microenvironment features of the tumor niche that enable cancer cells to reproduce characteristics such as differentiation, heterogeneity, proliferation, migration, and metabolism^39^. An example of this is a kind of spheroids, which are spherical 3D cultures developed from cell-cell interactions that drive the aggregation processes of cancer cells^18^. Usually developed from a single cell, spheroids are normally characterized by different organized areas that contribute to the reproduction of the tumor microenvironment^40^. The different oxygen and nutrient distribution within the spheroid induces an organized structure composed of an outer, actively proliferative layer and an inner apoptotic core^40,41^. Spheroids can also be originated from cell lines or from *ex vivo* primary cultures called cancer tissue-originated spheroids, which preserve the genetic and phenotypic features of a patient’s tumor^42,43^. Although these features make spheroids an ideal technology for studying the many aspects of a tumor, the lack of a complex 3D architecture limits their use to understand ECM-cancer interactions^44^. Organoids represent a different approach. Unlike cultures derived from single cells, organoids are tissue fragments cultured *in vitro* that maintain the heterogeneity and 3D spatial organization of the original cancer niche cell populations while also conserving the ECM structure and composition of the tumor of origin^44^. Thus, the nature of organoids endows this model with an important translational value, but it also confers some limitations. Surgical samples are often difficult to obtain by laboratories and their management is complex. In the present work, we successfully established a new 3D OSCC model using collagen-based scaffolds. Biomimetic porous scaffolds composed of biologically derived matrices are simple, inexpensive models for mimicking the tissue structure, tumor–stroma interactions, and structural/physical support of cancer cells^18^. Conversely, the biochemical and physical properties of the material (i.e., biocompatibility, porosity, and stiffness) partially condition cell behavior and the reproducibility of the structure. Our aim was to understand whether these 3D cultures can successfully reproduce the different features of HPV-positive and HPV-negative OSCC cells.

We demonstrated that the 3D structure affected cell growth dynamics and that the analyzed cell lines showed a different collagen fiber organization. Monolayer cultures displayed the typical proliferation rate characterized by an exponential growing phase, whereas 3D cultures showed different proliferation dynamics, resulting in a proliferation rate that remained virtually unaltered over time. Confocal imaging revealed a difference in the proliferation behavior between the core and the edge of the scaffold, e.g., 5 days after seeding, there was a higher proportion of cells from both cell lines in the edge than in the core area. The edge of the collagen matrix has closer contact with media nutrients and oxygen than the inner parts, and this microenvironment characteristic may partly explain the different proliferation rates between the 2 scaffold districts. Moreover, the cell distribution along the collagen fibers differed substantially in the 2 cell lines. HPV-negative cells showed a spindle-shaped mesenchymal phenotype and an aligned fiber distribution. These features are known as Indian filing, a morphology pattern often associated with aggressive invasive lobular breast carcinoma^33^. Conversely, HPV-positive cells showed an epithelial-like phenotype, growing in clusters that occupied much of the pore surface. We previously studied the alterations induced by 3D scaffolds in 2 breast cancer cell lines with different levels of aggressiveness^19^. The cells associated with high invasiveness displayed the same Indian file organization as that of HPV-negative cells, whereas less aggressive breast cancer cells and HPV-positive cell lines had similar epithelial-like morphologies and cell distribution. Overall, these results suggest that collagen-based scaffolds are capable of reproducing morphology features driven by cell aggressiveness that cannot be recreated by monolayer supports.

To better characterize our 3D model, we studied the influence of scaffolds on different pathways and processes involved in cancer progression. First, we investigated whether EMT processes play a role in the different behavior of the 2 cell lines. We analyzed the expression of *SNAIL* and *VIM*, 2 genes associated with mesenchymal phenotype^45,46^. During the culture period, *SNAIL* expression increased in both cell lines, whereas VIM expression was only upregulated in UM-SCC6. The 2 cell lines interacted differently with the 3D microenvironment, activating EMT pathways when seeded on scaffolds. These data provide evidence of the ability of our 3D model to induce a more mesenchymal phenotype than 2D cultures.

Then, our 3D model enabled us to study the interaction between cancer cells and the ECM, which affects several cancer processes^47^. This is not normally possible with 2D *in vitro* models. We used our 3D device to mimic the hierarchically organized ECM structure. Collagen, which is present in all human tissues, stimulates intracellular signaling through the activation of cell receptors and provides physical support for cell migration and proliferation^48^. We thus evaluated the expression of genes involved in the remodeling of the ECM: *MMP2*, *MMP9*, and *LOX*. MMPs are a group of endopeptidases responsible for modifying extracellular components^49^. Their interaction with scaffold collagen fibers induced MMP overexpression in the HPV-negative OSCC cell line but not in HPV-positive UPCI:SCC090. Conversely, the expression of *LOX*, an enzyme involved in ECM remodeling through the crosslinking of collagen and elastin, increased over time in the 3D cultures of both cell lines^50^. The interaction of OSCC cells with collagen fibers allowed this crosslinking activity. ECM is the product of several proteins that differ from tissue to tissue and from tumor to tumor^51,52^. Type I collagen represents a key structural component of ECM, influencing tumor invasion and progression^53^. It is composed of a 2-chain heterotrimer of collagen type I α1 and collagen type I α2, encoded by *COL1A1* and *COL1A2*, respectively^54^. We analyzed the TCGA and GTEx project databases using the Human Protein Atlas (https://www.proteinatlas.org/about) and GEPIA2^55^ (**Supplementary Figure S1A and S1B**) tools, identifying cancers expressing high levels of *COL1A1* and *COL1A2*. Head and neck cancer, breast cancer, and sarcomas are all malignancies with increased type I collagen expression. Our model could thus represent a suitable tool to study the role of the ECM in tumors with a high type I collagen content, including head and neck cancers. It could also facilitate the *in vitro* evaluation of cancer cell–ECM interactions in OSCC pathophysiology, which standard *in vitro* models cannot reproduce. However, its reliability in other cancer types warrants further investigation.

We looked at the potential involvement of hypoxia signaling in our 3D model as the development of a hypoxic microenvironment is one of the causes of altered growth dynamics and phenotypes. HIF-1α expression was observed in 3D in the nuclei along the whole scaffold surface. The hypoxia phenotype was associated with a change in cancer cell metabolism, with a switch toward glycolysis as a means of adapting to low oxygen levels^56^. GAPDH upregulation was observed in HPV-positive cells, and a high GLUT-1 expression was detected in both cell lines with respect to 2D monolayer cultures. These findings suggest that the hypoxic microenvironment could, in part, be responsible for phenotype alterations and drug resistance.

We used the zebrafish embryo model to confirm the aggressiveness of the phenotype acquired by OSCC cell lines grown in 3D scaffolds. In recent years, the number of available transgenic zebrafish strains has increased, offering new *in vivo* systems for several applications^18^. The transgenic strain Tg(fli1:EGFP) has green fluorescent blood vessels and represents a suitable model for the study of cancer migration processes. As far as we know, this is the first time that OSCC cells grown on biomimetic scaffolds have been injected into zebrafish. At 24 hpi, a higher number of embryos showed diffuse micrometastases when injected with scaffold cells with respect to 2D culture cells. In agreement with our *in vitro* results, the scaffolds increased the migration capability of OSCC cells.

Zhang et al.^57^ previously showed that the 3D structure of a polyester-based scaffold reduced the radiosensitivity of OSCC cell lines. Conversely, we investigated the sensitivity of our different biomimetic scaffold cultures to standard chemotherapeutic agents used to treat OSCC. Significant drug resistance to all treatments was observed in the 3D model compared with monolayer cultures, with the exception of CIS and CETU in UPCI:SCC090 cells. The cytotoxic effect of higher concentrations of CIS and CETU requires further investigation to elucidate the scaffold implication in the drug responses in these 2 conditions. HPV status is a strong and independent prognostic factor^6^. Patients with HPV-positive OSCC have a good prognosis, and their risk of death is half that of HPV-negative individuals^58^. Comparing the response to single treatments in monolayer cultures, the HPV-negative cell line exhibited higher sensitivity to 5-FU, CETU (*P* = 3.2 × 10^−5^), and GEM than the HPV-positive line UPCI:SCC090 (**Supplementary Figure S2**). Conversely, HPV-positive cells in 3D scaffolds were more sensitive to drugs than HPV-negative cultures: CIS (*P* = 0.004), 5-FU, CETU (*P* = 0.002), and GEM (*P* = 0.006). Overall, these results suggest that the biomimetic scaffold is capable of reproducing the response to drug regimens seen in real-life HPV-positive and HPV-negative patients. However, further investigation is needed to confirm this potentially important finding.

We assessed whether some of the mechanisms known to be involved in drug resistance, e.g., *NT5E*, *FOXM1*, *FLT1*, and *ABCA3*, are modulated in 3D compared with standard monolayer cultures^59–64^. *NT5E* and *FOXM1* genes encode for glycosylphosphatidylinositol-anchored cell surface protein and oncogenic transcription factor Forkhead box protein M1, respectively. *FLT1* encodes for vascular endothelial growth factor receptor 1, a gene associated with anti-EGFR drug resistance^50^. *ABCA3* is a member of the ATP-binding cassette family, which includes transporters driving chemotherapeutic agents outside the cell^64^. In our study, mRNA relative quantification highlighted a different expression pattern in the 2 culture systems. Both cell lines grown in 3D showed increased *NT5E* expression, albeit with different modulations, whereas only UPCI:SCC090 overexpressed *FOXM1*. Conversely, UM-SCC6 showed an upregulation of *FLT1* and *ABCA3* when seeded in scaffolds, but this was not observed in UPCI:SCC090. These findings suggest that collagen-based scaffolds are capable of activating drug-resistance mechanisms but *via* different pathways and depending on the cell type. For example, *FLT1*, which is associated with anti-EGFR drug resistance, was upregulated in HPV-negative UM-SCC6 and downregulated in HPV-positive UPCI:SCC090 3D cultures. CETU, an EGFR inhibitor, induced a different effect on UM-SCC6 2D and 3D systems, but not on the 2 UPCI:SCC090 culture types. Thus, CETU effects on our scaffold cultures match with the expression of *FLT1*.

We used patient-derived primary cultures to confirm the ability of collagen-based scaffolds to induce drug resistance. Immortalized cell lines are cells that have been manipulated to proliferate indefinitely, and a prolonged length of time in an *in vitro* environment contributes to altering the original nature of the cell population. *Ex vivo* samples have the advantage of preserving many of the original features of the cancer lesion that are essential for the reproduction of the tumor microenvironment^18^. We used, for the first time, primary cultures and collagen-based scaffold devices to investigate the impact of chemotherapy treatments in head and neck cancers. The impact of drug treatments on 2D and 3D primary cultures was similar to that on cell lines. Primary cultures conserve part of the stromal populations and different subclones of patient tumor tissue. The crosstalk between healthy and malignant components plays a role in the induction of drug resistance^16,18^. In this scenario, the modest differences between primary cultures and cell lines might be ascribable to the presence of stromal cells. However, further analysis is needed to better understand the behavior of primary cultures in collagen-based scaffolds.

The above findings indicate the substantial influence exerted by the 3D structure on the phenotype and genotype of cancer cells. The alterations seem to be different for each cancer cell type but might be oriented by the HPV status of the tumor. HPV-negative UM-SCC6 cells in 3D cultures acquired a spindle-shaped morphology and showed an aligned distribution of fibers, overexpresion of EMT-related genes, and enhanced *in vitro* and *in vivo* migration ability. Some of these features were also induced in HPV-positive UPCI:SCC090 cells, but with substantial differences. HPV-positive cells grew in clusters that filled up scaffold pores, acquired an epithelial-like phenotype, expressed low levels of MMPs, and showed less accentuated migration behavior than HPV-negative cells. Conversely, both cell lines on 3D scaffolds were less sensitive to treatments but activated the expression of different drug-resistance markers. In conclusion, the induction of migration properties and of EMT marker expression are 2 biological characteristics associated with cancer aggressiveness. Our results thus suggest that 3D scaffolds are capable of reproducing the different nature of HPV-related malignancies.

## Conclusions

Our biomimetic scaffold proved to be a simple and cost-effective model to study the interactions between ECM and cancer cells. It is also the first study using zebrafish models to investigate the behavior of HPV-positive and HPV-negative OSCC cancer cells and to compare the aggressiveness induced by 3D collagen-based scaffold. The characterization of HPV-positive and HPV-negative cell lines in our 3D cultures underlined the different pathophysiological nature and drug sensitivity of these 2 tumor types. Overall, our results confirm the ability of 3D scaffolds to reproduce the microenvironment features of OSCC that are difficult to study with other *in vitro* systems.
