# Supplementary material for: Three-dimensional collagen-based scaffold model to study the microenvironment and drug-resistance mechanisms of oropharyngeal squamous cell carcinomas
Source: Cancer Biol Med. 2021 Jun 15;18(2):502–16. doi: 10.20892/j.issn.2095-3941.2020.0482 (PMC8185858; doi:10.20892/j.issn.2095-3941.2020.0482)
Supplement: Supplementary file 1 [file cbm-18-502-s001.pdf]

Supplementary materials

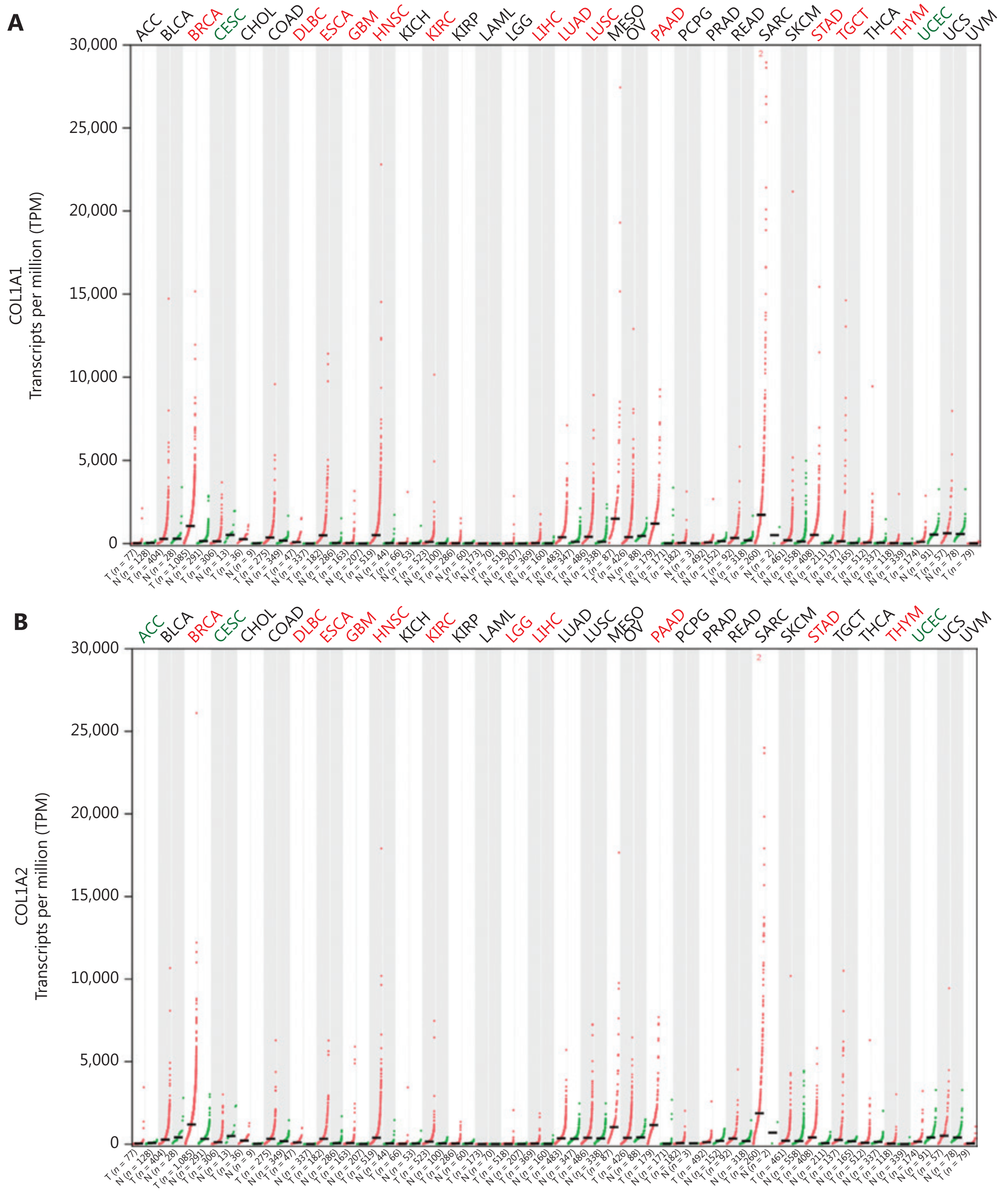

Figure S1 (A, B) *COL1A1* and *COL1A2* gene expression profiles across tumor samples (T) and paired healthy tissue (N). Gene expression profiles were obtained using the GEPIA2 web server. ACC, adrenocortical carcinoma; BLCA, urothelial bladder carcinoma; BRCA, breast invasive carcinoma; CESC, cervical squamous cell carcinoma and endocervical adenocarcinoma; CHOL, cholangiocarcinoma; COAD, colon adenocarcinoma; DLBC, diffuse large B-cell lymphoma; ESCA, esophageal carcinoma; GBM, glioblastoma multiforme; HNSC, head and neck squamous cell carcinoma; KICH, kidney chromophobe; KIRC, kidney renal clear cell carcinoma; KIRP, kidney renal papillary cell carcinoma; LAML, acute myeloid leukemia; LGG, low-grade glioma; LIHC, liver hepatocellular carcinoma; LUAD, lung adenocarcinoma; LUSC, lung squamous cell carcinoma; MESO, mesothelioma; OV, ovarian serous cystadenocarcinoma; PAAD, pancreatic adenocarcinoma; PCPG, pheochromocytoma and

paraganglioma; PRAD, prostate adenocarcinoma; READ, rectum adenocarcinoma; SARC, sarcoma; SKCM, skin cutaneous melanoma; STAD, stomach adenocarcinoma; TGCT, testicular germ cell tumors; THCA, thyroid carcinoma; THYM, thymoma; UCEC, uterine corpus endometrial carcinoma; UCS, uterine carcinosarcoma; UVM, uveal melanoma.

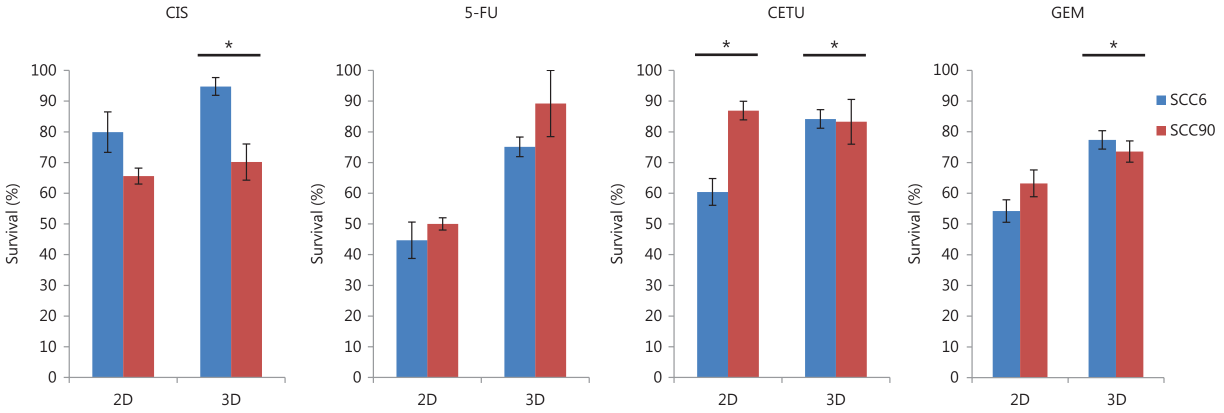

Figure S2 3D scaffold induces a higher drug response in HPV-positive cells compared to HPV-negative cultures. Cytotoxicity analysis of 2D and 3D UM-SCC6 and UPCI:SCC090 cultures treated with CIS, 5-FU, CETU, and GEM. Differences between 2D and 3D cultures were assessed by a 2-tailed Student's *t*-test and accepted as significant (*) at $P < 0.05$. HPV, human papilloma virus; 3D, 3-dimensional; 2D, 2-dimensional; CIS, cisplatin; 5-FU, 5-fluorouracil; CETU, cetuximab; GEM, gemcitabine.
